# *MMP9* Gene Polymorphism (rs3918242) Increases the Risk of Cardiovascular Disease in Type 2 Diabetes Patients

**DOI:** 10.3390/jcm12226990

**Published:** 2023-11-08

**Authors:** Monika Buraczynska, Sylwia Wrzos, Wojciech Zaluska

**Affiliations:** Department of Nephrology, Medical University of Lublin, 20-093 Lublin, Poland; sylwiawrzos@umlub.pl (S.W.); wojciech.zaluska@umlub.pl (W.Z.)

**Keywords:** *MMP9* gene, type 2 diabetes, cardiovascular disease, single nucleotide polymorphism, risk allele

## Abstract

Matrix metalloproteinase 9 (*MMP-9*) C(-1562)T gene polymorphism has been considered a risk factor for cardiovascular disease (CVD). Our study aimed to evaluate the association between this polymorphism and CVD in diabetes patients. The genotyping was performed in 740 patients with T2DM and 400 healthy subjects. A significant difference in the polymorphism distribution was revealed between patients and controls. The T allele and TT homozygote were associated with increased risk of diabetes (OR 1.88, *p* < 0.0001 and OR 3.77, *p* = 0.0002, respectively). The comparison between CVD+ and CVD− subgroups showed a much higher frequency of the T allele in patients with CVD (OR 2.87, 95% CI 2.14–3.85, *p* < 0.0001). Patients with the TT genotype had a higher prevalence of CVD (OR 3.19, 95% CI 1.55–6.56, *p* = 0.0015). The carrier genotypes (CT/TT) were correlated with HDL levels in both CVD+ and CVD− subgroups (*p* < 0.001 for both). In the logistic regression analysis, only C(-1562)T SNP was a significant predictor of CVD in diabetic patients (*p* < 0.001). In conclusion, our study suggests an association between *MMP-9* C(-1562)T polymorphism and an increased risk of CVD in T2DM. If replicated in other studies, it could be considered a genetic marker for predicting risk of T2DM and its cardiovascular comorbidity.

## 1. Introduction

Type 2 diabetes mellitus (T2DM) is a chronic, heterogenous disorder of glucose homeostasis presenting with persistent hyperglycemia. It arises from insufficient insulin secretion and/or insulin resistance. T2DM constitutes close to 90% of diabetes cases and contributes to the burden on health care system [1]. This multifactorial condition is associated with high morbidity and mortality due to macrovascular and microvascular complications. The presence of cardiovascular disease (CVD) in patients with type 2 diabetes is a serious clinical problem. CVD is one of the most frequent causes of death among DM patients. It accounts for approximately 65 % of mortality in DM [2].

Matrix metalloproteinases (MMPs) are a large group of zinc-dependent endopeptidases that degrade various components of the extracellular matrix in both physiological and pathological processes [3,4]. They are synthesized and released in the form of pro-proteins that need to undergo a cleaving process to become biologically active. MMPs are engaged in the pathogenesis of atherosclerosis by promoting the migration, proliferation and differentiation of smooth muscle cells. They also induce and destabilize atherosclerotic plaques. The actions of metalloproteinases are controlled by the mechanisms of transcription and translation as well as by inhibitors [5,6]. The altered expression and dysregulated activity of MMPs have been observed in clinical conditions involving disorders of the musculoskeletal system, inflammatory processes and cardiovascular and cerebrovascular diseases [6,7,8,9].

Matrix metalloproteinase 9 (MMP9) (gelatinase B) has a proteolytic activity against proteins of connective tissue, mainly collagens, proteoglycans and elastin. It is highly expressed in atherosclerotic plaques and involved in plaque progression and rupture [10]. Elevated levels of MMP9 have been reported in coronary artery disease (CAD) and hypertension and are also related to cardiovascular events and mortality [11,12,13,14,15].

The human *MMP9* gene is located in the chromosome region 20q11.2-q13.1 [16] and contains several single nucleotide polymorphisms. Genetic variants in the regulatory regions of the *MMP9* gene are functionally important and modify levels of the protein [16,17,18]. The *MMP9*-1562 C/T, a single nucleotide polymorphism, is located in the promoter region of the *MMP9* gene. It was reported to exert a functional allele-specific effect on gene transcription. A C-to-T substitution causes the loss of nuclear protein binding to the region and a rise in the transcriptional activity in macrophages. The CC genotype is associated with the decreased activity of promoter, whereas the T allele (CT and TT genotypes) induces higher transcriptional activity [16]. The -1562 C/T variant has been extensively studied and the T allele has been reported to be associated with elevated circulating MMP levels, cardiovascular disease and arterial stiffness [17,19,20,21]. *MMP9* -1562 T allele carriers were predisposed to coronary atherosclerosis and had an increased severity of the disease [16]. This polymorphism was also reported to be associated with microvascular complications in diabetes [22]. We hypothesize that the minor allele of the *MMP-9* -1562 C/T SNP is also associated with cardiovascular disease in subjects with T2DM.

Since the literature on the increased genetic susceptibility to CVD in diabetes patients is rather scarce, in this study we aimed to investigate an association between the -1562 C/T polymorphism in the *MMP9* gene and the risk of cardiovascular disease in subjects with T2DM.

## 2. Materials and Methods

### 2.1. Study Subjects

All patients enrolled in this retrospective case–control study were recruited from University Hospital, Medical University of Lublin. The patient group included 740 unrelated subjects (392 males and 348 females) with type 2 diabetes of at least 10 years duration (mean age 57.2 ± 9.1 years). All patients were Caucasians of Polish origin. The same patient cohort and control groups were used as described in detail in our earlier study [23].

The diagnosis of type 2 diabetes was based on the American Diabetes Association criteria [24]. A complete physical examination of all subjects included plasma fasting glucose, glycated hemoglobin (HbA1c), full lipid profile, albumin-to-creatinine ratio (ACR), albumin excretion rate (AER) and body mass index (BMI) measurements.

The following exclusion criteria were applied: type 1 and other types of diabetes, acute or chronic inflammatory conditions, autoimmune diseases and history of malignancies.

Cardiovascular disease was diagnosed in 522 patients (70.5%). They had one or a combination of pathological phenotypes: congestive heart failure, left ventricular hypertrophy, angina pectoris, ischemic heart disease, myocardial infarction and ischemic cerebral stroke. The clinical presentation of CVD was verified by relevant biochemical tests, as well as the radiographic, echocardiographic and vascular diagnostic criteria.

The healthy control group of 400 individuals (mean age 57.5 ± 8.1 years) consisted of unrelated volunteers with no known history of diabetes and cardiovascular disease.

Participation in the study was voluntary and before being included all T2DM patients and healthy controls provided a written informed consent, in accordance with principles of the Declaration of Helsinki (version 2013). The ethical approval of the detailed research protocol for the study was provided by the Bioethics Committee of the Medical University of Lublin (KE-0254/26/01/2023).

### 2.2. Determination of MMP9 rs3918242 (-1562 C/T) Genotype

After collecting 5 mL of peripheral venous blood from all enrolled individuals, genomic DNA was extracted from leukocytes via the standard procedure. The DNA concentration and its purity were determined using a Nano Drop 2000 (Thermo Scientific, Waltham, MA, USA). DNA samples were stored at −70 °C before use. The rs3918242 variant in the *MMP9* gene was detected with amplification of the 435 bp DNA target via a polymerase chain reaction (PCR) assay. The following sequence-specific primers were used for DNA amplification: the sense primer 5′-GCCTGGCACATAGTAGGCCC-3′ and antisense primer 5′-CTTCCTAGCCAGCCGGCATC-3′, as reported earlier [25]. The conditions for the PCR reactions were slightly modified, starting with an initial denaturation step at 95 °C for 5 min, followed by 35 cycles of denaturation at 95 °C for 30 s, annealing at 65 °C for 1 min and extension at 72 °C for 1 min. The length of the PCR product was 435 bp. The polymorphism was detected by digesting amplicons with Sph I restriction endonuclease (Thermo Scientific) and separating DNA fragments on a 2% agarose gel. The C allele showed as an undigested 435 bp fragment, whereas the polymorphic variant (T allele) produced two fragments of 247 bp and 188 bp. The case/control status of the DNA samples was blinded for genotyping. The validation of genotypes was performed by repeating PCR amplification in 20% of random samples. In addition, 20 samples for each genotype were re-analyzed via sequencing using the CEQ 8000 Genetic Analysis System (Beckman Coulter, England) for the correctness of genotype reading in agarose gel. A 100% concordance was achieved between the genotyping assays.

### 2.3. Statistical Analysis

Statistical analysis of the results was conducted using SPSS software (version 19) (SPSS, Inc., Chicago, IL, USA). In descriptive statistics for the differences in the baseline parameters between the case and control groups, the normally distributed quantitative data are shown as means ± SD. Categorical variables are represented as numbers and percentages. The frequencies of alleles and genotypes were obtained by direct counting. The potential deviation in genotype distribution from the Hardy–Weinberg equilibrium (HWE) was assessed with the chi-squared test. The genotype/allele frequencies were compared between groups (T2DM patients and controls) and subgroups (CVD+ and CVD− patients) using a chi-squared test of independence with 2 × 2 contingency and *z* statistics. For comparison of continuous and categorical variables between groups, we used the *t* test and Pearson’s Χ^2^ test of independence. The odds ratios (OR) with corresponding 95% confidence intervals (CI) were reported for associations. ORs were adjusted for age, gender, lipid parameters and T2DM duration. Unconditional logistic regression analysis was carried out for confirming the rs3918242 association with disease and interaction with other risk factors. A two-tailed type I error rate of 5% was regarded as statistically significant.

## 3. Results

### 3.1. Subject Characteristics

The genotype of the rs3918242 SNP in the *MMP9* gene was determined in a total of 740 patients with T2DM and 400 healthy control subjects. The success rate for genotyping was 100%. The minor allele frequency was similar to that reported in another Polish study [26]. The comparison of the demographic features and clinical and laboratory parameters of diabetic patients and controls is presented in Table 1. In a group of 740 patients with T2DM, 522 (70.5%) had cardiovascular disease. All parameters were compared between this subgroup (CVD+) and 218 T2DM patients without CVD (CVD−). The gender distribution was similar in patients with CVD and without CVD (*p* = 0.082). On the other hand, patients with CVD were older than those without it (*p* < 0.001). A statistically significant difference was also observed in the age at diagnosis of T2DM (*p* = 0.007) and disease duration (*p* < 0.001). As expected, there was a significant difference in the levels of total cholesterol, HDL cholesterol and triglyceride between the CVD+ and CVD− subgroups. Significant differences between T2DM patients and healthy controls were seen in age, total cholesterol level and BMI (*p* < 0.001 for all variables).

### 3.2. Genotyping Analysis

The genotyping results of the rs3918242 SNP in the *MMP-9* gene in patients and controls are presented in Table 2. The genotype frequencies in the T2DM and control groups were as predicted by the Hardy–Weinberg equilibrium (*p* = 0.259 for patients and *p* = 0.580 for controls). In the comparison of the polymorphism distribution in the T2DM and control groups, we observed a statistically significant difference. The T allele and TT homozygote were associated with an increased risk of T2DM (OR 1.88, 95% CI 1.51–2.34, *p* < 0.0001 and OR 3.77, 95%CI 1.88–7.56, *p* = 0.0002, respectively).

### 3.3. Association between -1562 C/T SNP and the Risk of Cardiovascular Disease

All T2DM patients were stratified into CVD+ (*n* = 522) and CVD− (*n* = 218) subgroups for further comparisons (Table 2). The frequencies of the T allele and TT genotype were significantly increased in T2DM CVD+ patients compared with those without CVD. The odds ratio for the T allele was 2.87, 95% CI 2.14–3.85 (*p* < 0.0001) and for the TT genotype it was 3.19, 95% CI 1.55–6.56 (*p* = 0.0015). This indicates that the risk of developing CVD in the carriers of the T allele was three-fold higher than in non-carriers. As presented in Table 3, there was no significant association observed between the *MMP-9* rs3918242 polymorphism and CVD risk using the recessive genetic model (OR 1.82, 95% CI 0.89–3.69, *p* = 0.0976). Overall, the results show statistically significant association in two genetic models, codominant (Table 2) and dominant (Table 3), with OR 2.87, 95% CI 2.14–3.85, *p* < 0.0001 and OR 4.26, 95%CI 3.0–6.06, *p* < 0.0001, respectively).

Table 4 shows the CVD-associated parameters according to the carrier and non-carrier genotypes of *MMP-9* rs3918242 polymorphism. The carrier genotype (CT/TT) was correlated with the HDL level in both the CVD+ and CVD− subgroups (*p* < 0.001 and *p* = 0.005, respectively). The other parameters did not show any significant association with genotype.

Multiple logistic regression analysis was carried out to confirm the independent risk factors for CVD in diabetes patients (Table 5). It was found that only *MMP-9* rs3918242 polymorphism and diabetic retinopathy were significant risk predictors for CVD development in T2DM patients (*p* < 0.001 and 0.028, respectively).

## 4. Discussion

The presence of cardiovascular disease in type 2 diabetes patients is a serious clinical problem [27]. Matrix metalloproteinase MMP-9 is of particular importance in the development of CVD as it is expressed in numerous cardiovascular cell types, mainly in cardiomyocytes, cardiac fibroblasts, vascular endothelial cells and inflammatory cells [6]. Several studies have documented MMP-9’s contribution to CVD in individuals with diabetes.

In this study, we assessed the association between the -1562 C/T polymorphism (rs3918242) in the *MMP9* gene and the susceptibility to cardiovascular disease in T2DM patients. This SNP was selected based on earlier association studies in cardiovascular diseases. The previous reports demonstrated that this polymorphism is associated with diabetes. Along with a direct impact on type 2 diabetes, studies have also reported links to metabolic syndrome and complications associated with T2DM. It has possible effects on diabetic nephropathy, retinopathy, diabetic foot and macroangiopathy [22,28,29]. In our study, the minor T allele carriership was associated with type 2 diabetes. A total of 49% of individuals were T allele carriers in the T2DM patient group compared with 31.5% in the control group (*p* < 0.0001). The risk of T2DM for the T allele carriers was over two-fold higher than for non-carriers.

For the analysis of an association between the -1562 C/T SNP and cardiovascular disease, the distribution of the polymorphism was compared in the CVD+ and CVD− subgroups of T2DM patients. A strong association between the T allele and TT homozygous genotype and the occurrence of cardiovascular disease was observed in this analysis in two genetic models (co-dominant and dominant). The increased frequency of T allele carriers in the T2DM CVD+ subgroup supports the role of the T allele as a genetic susceptibility factor for CVD. Earlier, Buraczynska et al. studied the association of the *MMP-9* -1562 C/T gene polymorphism with the risk of stroke in 322 Polish patients (52% of patients had type 2 diabetes). Their findings suggested that -1562C/T genotypes are significantly associated with the ischemic stroke risk in patients with and without diabetes. They concluded that although the observed effect on susceptibility to stroke is modest, it might be enhanced by interaction with other genetic factors [30]. The association of the *MMP-9* -1562 C/T SNP with CVD was also found in another Polish study of 110 subjects with coronary atherosclerosis. The results indicated that C to T transition is associated with premature ischemic heart disease in the studied cohort [31].

The same polymorphism was investigated in relation to cardiovascular diseases in other European studies. Watson et al. evaluated the association of the rs3918242 *MMP-9* gene polymorphism (-1562 C/T) with hypertension, myocardial infarction and ventricular dysfunction in Irish patients with diabetes. In a cohort of 498 T2DM patients, they observed a two-fold higher risk of myocardial infarction and a lower ejection fraction (OR2.56, *p* = 0.026) in the carriers of the minor T allele of this polymorphism [32]. These data might be important in relation to the burden of heart failure in diabetic patients and emphasize the potential of MMP-9 polymorphism as a biomarker [32]. In a cohort of 1000 Norwegian patients with angiographically verified stable CAD and metabolic syndrome, the authors analyzed the effect of the -1562 C/T polymorphism on the clinical events after a 2-year follow-up. They observed a five-fold increase in the risk of clinical events in the T allele carriers of the -1562 C/T polymorphism. This effect was probably mediated through elevated MMP-9 levels and altered regulation of MMP-9 [33]. Blankenberg et al. performed a prospective study of 1127 patients with CAD, analyzing MMP-9 levels as a novel predictor of CV mortality and the genotypes of two *MMP-9* gene polymorphisms: -1562 C/T and R279Q. The T allele of the -1562 C/T SNP was associated with increased MMP-9 levels. The R279Q polymorphism had no direct effect on MMP-9 concentration but was associated with CV events in patients with stable angina [17]. Boshev et al. studied the rs3918242 polymorphism in a younger population with CAD and concluded that this polymorphism could be used for clinical risk assessment for the development of CAD [34].

Reports from other populations also indicate that the *MMP-9* gene -1562 C/T polymorphism is associated with the risk of cardiovascular disease. In a study of 261 Chinese patients with coronary artery disease, the authors investigated the correlation of the -1562 C/T polymorphism and the susceptibility to CAD. The TT and CT genotypes of this polymorphism were strongly associated with a raised risk of coronary artery disease [35]. A study of 236 Mexican patients with a history of myocardial infarction revealed an association between a functional -1562 C/T promoter region polymorphism and the risk of developing MI. The risk for the carriers of the T allele was almost three-fold higher than for patients with the CC genotype. According to the authors, the results of their study support the role of this polymorphism as a genetic marker for susceptibility to MI [21]. Similarly, the -1562 C/T polymorphism was found to be strongly associated with MI in another study involving 206 patients with acute myocardial infarction [20]. Some studies have reported contradictory results. In a study of 1287 individuals with myocardial infarction (854 patients) and stroke (367 patients), the *MMP-9* SNPs were not significantly associated with MI or stroke [36]. In a study by Mahmoodi et al. on 100 patients with coronary artery disease, -1562 C/T polymorphism in the *MMP-9* gene was not associated with CAD [37]. In an Indian study of young patients with ST-segment elevation MI, no relationship was found between -1562 C/T polymorphism and the disease [38]. These discrepancies between studies might be related to the different genetic backgrounds in the studied populations or to variations in the study design or selection criteria for the patients and controls.

The exact mechanism of the effect of the *MMP-9* -1562 C/T polymorphism in the promoter region of the gene on the susceptibility to cardiovascular disease is unknown. It was found that the transcriptional activity of the T allele was significantly increased compared with the activity of the wild-type C allele [19,39]. Thus, increased expression levels of the *MMP-9* gene may have an atherogenic effect. It was reported from autopsy studies that the aortic and coronary arteries of diabetic patients showed higher expression of MMP-9 compared with patients without diabetes. The MMP-9 levels were correlated with HbA1c and apoptosis [40]. In diabetes patients, chronic low-grade inflammation can cause variations in extracellular matrix turnover and adverse changes in myocardial tissues [41].

Our study has certain limitations. Although we applied strict selection criteria to define our patient and control groups, there was a potential selection bias due to the design of the study being retrospective. Patients were enrolled regardless of the time of CVD diagnosis. It is possible that some of the T2DM patients classified as CVD− could have undetected subclinical atherosclerosis that could affect the results. Furthermore, our study was restricted to one SNP in the *MMP-9* gene, so the effect of other polymorphisms at this locus on the development of CVD cannot be excluded. The other limitation is that the functional consequences of the rs3918242 polymorphism were not investigated in our study.

In conclusion, the results of our study suggest a strong association between the *MMP-9* -1562 C/T (rs3918242) gene polymorphism and an increased risk of cardiovascular disease in type 2 diabetes patients. The polymorphism can be considered as a potential marker for predicting the risk of type 2 diabetes and cardiovascular comorbidities/complications in diabetic patients. It is novel, clinically relevant information and, if replicated in a larger cohort of T2DM patients, could play an important role in personalized medicine.

## Figures and Tables

**Table 1:** Comparison of the clinical and laboratory characteristics of T2DM patients and healthy controls.

Variables	T2DM Patients	CVD+	CVD−	Healthy Controls	*p* Value *
N	740	522	218	400	
Gender (male/female)	392/348	287/235	105/113	205/195	0.082
Age (years)	60.2 ± 9.4	63.6 ± 9.1	56.8 ± 9.6	57.5 ± 8.1	<0.001
Age at diabetes diagnosis (years)	43.2 ± 7.6	42.4 ± 7.1	44.0 ± 8.1	NA	0.007
Diabetes duration (years)	15.0 ± 9.3	16.7 ± 8.3	13.3 ± 9.3	NA	<0.001
Total cholesterol (mmol/L)	4.8 ± 1.2	4.9 ± 1.3	4.7 ± 1.0	4.0 ± 0.8	0.042
HDL cholesterol (mmol/L)	1.21 ± 0.31	1.1 ± 0.2	1.3 ± 0.2	ND	<0.001
Triglyceride (mmol/L)	2.1 ± 0.9	2.3 ± 0.8	1.9 ± 0.8	ND	<0.001
HbA_1c_ (%)	8.2 ± 2.5	8.3 ± 1.9	8.1 ± 3.1	ND	0.285
BMI (kg/m^2^)	29.8 ± 8.8	30.4 ± 8.6	29.3. ± 9.0	27.1 ± 4.2	0.118

T2DM, type 2 diabetes mellitus; CVD+, those with cardiovascular disease; CVD−, those without cardiovascular disease; BMI, body mass index; HbA_1c_, glycated hemoglobin; NA, not applicable. ND, not determined. Data are reported as means ± SD or numbers and percentages (in parentheses). * *p* calculated for CVD+ vs. CVD−. In a comparison between the T2DM and control groups, statistically significant *p*-values were observed in age, total cholesterol and BMI (for all *p* < 0.001).

**Table 2:** Genotype and allele distribution of *MMP-9* rs3918242 polymorphism in patients with T2DM and healthy controls.

		Genotypes			MAF	OR (95% CI) ^b^	
	N	CC	CT	TT		T Allele	TT Genotype ^a^
T2DM	740	377 (51)	311 (42)	52 (7)	0.28	1.88 (1.51–2.34)	3.77 (1.88–7.56)
						*p* < 0.0001	*p* = 0.0002
T2DM CVD+	522	214 (41)	266 (51)	42 (8)	0.34	2.87 (2.14–3.85)	3.19 (1.55–6.56)
						*p* < 0.0001	*p* = 0.0015
T2DM CVD−	218	163 (74)	45 (21)	10 (5)	0.15	Ref. for T2DM CVD+	
Controls	400	274 (68.5)	116 (29)	10 (2.5)	0.17	Ref. for T2DM	

T2DM, type 2 diabetes mellitus; T2DM CVD+, T2DM with cardiovascular disease; T2DM CVD−, T2DM without CVD. Genotype distribution is shown as numbers with percentages in parenthesis. ^a^ Calculated versus CC genotype. Hardy–Weinberg equilibrium: χ^2^ = 1.269, *p* = 0.259 for the T2DM group; χ^2^ = 0.305, *p* = 0.580 for the control group. ^b^ ORs adjusted for age, gender, lipid parameters and T2DM duration: T2DM vs. C: T allele 1.66 (1.31–2.43), *p* < 0.001; TT genotype 3.26 (1.72–8.31), *p* < 0.001; CVD+ vs. CVD−: T allele 3.14 (1.45–7.12), *p* < 0.001; TT genotype 2.96 (1.65–3.94), *p* < 0.001.

**Table 3:** Distribution of the *MMP-9* rs3918242 polymorphism according to dominant and recessive genetic models.

*MMP-9* rs3918242	T2DM CVD+	T2DM CVD−	OR (95% CI)	*p* Value
C/T Genotypes	(*n* = 522)	(*n* = 218)		
Dominant model				
CC	214 (41)	163 (75)	ref.	
CT + TT	308 (59)	55 (25)	4.26 (3.0–6.06) ^a^	<0.0001
Recessive model				
CC + CT	480 (92)	208 (95)	ref.	
TT	42 (8)	10 (5)	1.82 (0.89–3.69) ^b^	0.0976

*MMP-9*, matrix metalloproteinase 9 gene. T2DM, type 2 diabetes mellitus. Genotype distribution is shown as numbers with percentages in parenthesis. Odds ratio (OR) is related to the ^a^ CC homozygote and ^b^ CC + CT genotypes.

**Table 4:** CVD-associated parameters according to the carrier and non-carrier genotypes of *MMP-9* rs3918242 polymorphism.

Parameters	T2DM CVD+ (*n* = 522)			T2DM CVD− (*n* = 218)		
	CC	CT + TT	*p*-Value	CC	CT + TT	*p*-Value
Age (years)	63.4 ± 8.6	63.8 ± 9.6	0.625	56.9 ± 9.1	56.7 ± 10.1	0.891
TC (mmol/L)	4.9 ± 1.6	4.9 ± 1.0	1.000	4.6 ± 1.3	4.8 ± 0.7	0.277
TG (mmol/L)	2.3 ± 0.2	2.3 ± 1.3	1.000	1.9 ± 1.0	1.8 ± 0.6	0.484
HDL (mmol/L)	1.2 ± 0.1	1.0 ± 0.2	<0.001	1.3 ± 0.2	1.2 ± 0.3	0.005
SBP (mmHg)	145 ± 20.2	147 ± 18.5	0.242	145 ± 17.6	143 ± 19.3	0.477
DBP (mmHg)	83 ± 11.2	82 ± 9.6	0.509	85 ± 14.6	83 ± 10.3	0.348
DBP (mmHg)	29.9 ± 6.8	30.9 ± 10.4	0.217	28.8 ± 13.2	29.8 ± 4.8	0.583

T2DM, type 2 diabetes mellitus; T2DM CVD+, T2DM with cardiovascular disease. T2DM CVD−, T2DM without CVD. TC, total cholesterol. TG, triglyceride. HDL, high density lipoprotein. BMI, body mass index. SBP, systolic blood pressure. DBP, diastolic blood pressure. For CVD+ non-carriers (CC), *n* = 214; for carriers (CT + TT), *n* = 308. For CVD− non-carriers (CC), *n* = 163; for carriers (CT + TT), *n* = 55.

**Table 5:** The results of multivariate logistic regression analysis for independent risk factors for CVD.

Included Variables	Adjusted Odds Ratio	95% CI	*p* Value
Age	1.21	0.58–1.42	0.086
Gender	1.62	0.85–1.93	0.072
T2DM duration	1.47	0.76–2.08	0.047
Age of onset	1.16	0.61–2.26	0.412
Hypertension	1.84	0.73–3.94	0.073
Diabetic retinopathy	2.53	1.62–4.23	0.028
Diabetic nephropathy	1.97	0.65–2.74	0.058
Diabetic foot	2.33	1.58–3.92	0.047
BMI	1.27	0.84–1.46	0.267
T allele *	2.48	1.86–4.62	<0.001

T2DM, type 2 diabetes mellitus. * T allele of *MMP-9* rs3918242 polymorphism. An unconditional model of multiple logistic regression analysis was used for the interaction between the *MMP-9* gene polymorphism and other variables.

## Data Availability

The data supporting the findings of this study are available on request from the corresponding author.

## References

[1] Cho N.H., Shaw J.E., Karuranga S., Huang Y., da Rocha Fernandes J.D., Ohlrogge A.W., Malanda B. (2018). IDF Diabetes Atlas: Global estimates of diabetes prevalence for 2017 and projections for 2045. Diabetes Res. Clin. Pract..

[2] Visaria J., Iyer N.N., Raval A., Kong S., Hobbs T., Bouchard J., Kern D.M., Willey V. (2019). Incidence and prevalence of microvascular and macrovascular diseases and all cause mortality in type 2 diabetes mellitus. A 10 year study in a US commercially insured and Medicare Advantage population. Clin. Ther..

[3] Visse R., Nagase H. (2003). Matrix metalloproteinases and tissue inhibitors of metalloproteinases: Structure, function and biochemistry. Circ. Res..

[4] Cui N., Hu M., Khalil R.A. (2017). Biochemical and biological attributes of matrix metalloproteinases. Prog. Mol. Biol. Transl. Sci..

[5] Gallis Z.S., Khatri J.J. (2002). Matrix metalloproteinases in vascular remodeling and atherogenesis: The good, the bad, and the ugly. Circ. Res..

[6] Simões G., Pereira T., Caseiro A. (2022). Matrix metaloproteinases in vascular pathology. Microvasc. Res..

[7] Siefert S.A., Sarkar R. (2012). Matrix metalloproteinases in vascular physiology and disease. Vascular.

[8] Chen Q., Jin M., Yang F., Zhu J., Xiao Q., Zhang L. (2013). Matrix metalloproteinases: Inflammatory regulators of cell behaviors in vascular formation and remodeling. Mediat. Inflamm..

[9] Mittal R., Patel A.P., Debs L.H., Nguyen D., Patel K., Grati M., Mittal J., Yan D., Chapagain P., Liu X.Z. (2016). Intricate functions of matrix metalloproteinases in physiological and pathological conditions. J. Cell. Physiol..

[10] Galis Z.S., Sukhova G.K., Lark M.W., Libby P. (1994). Increased expression of matrix metalloproteinases and matrix degrading activity in vulnerable regions of human atherosclerotic plaques. J. Clin. Investig..

[11] Tayebjee M.H., Lip G.Y., Tan K.T., Patel J.V., Hughes E.A., MacFadyen R.J. (2005). Plasma matrix metalloproteinase-9, tissue inhibitor of metalloproteinase-2, and CD-10 ligand levels in patients with stable coronary artery disease. Am. J. Cardiol..

[12] Yasmin, Wallace S., McEniery C.M., Dakham Z., Pusalkar P., Maki-Petaja K., Ashby M.J., Cockcroft J.R., Wilkinson I.B. (2005). Matrix metalloproteinase-9 (MMP-9), MMP-2 and serum elastase activity are associated with systolic hypertension and arterial stiffness. Arterioscler. Tromb. Vasc. Biol..

[13] Derosa G., D′Angelo A., Ciccarelli L., Piccinni M.N., Pricolo F., Salvadeo S., Montagna L., Gravina A., Ferrari I., Galli S. (2006). Matrix metalloproteinase-2, -9, and tissue inhibitor of metalloproteinase-1 in patients with hypertension. Endothelium.

[14] Furenes E.B., Trøseid M., Solheim S., Hjerkinn E.M., Seljeflot I., Arnesen H., Weiss T.W. (2010). Prediction of cardiovascular events by matrix metalloproteinase (MMP-9) in elderly men. Tromb. Haemost..

[15] Wang X., Khalil R.A. (2018). Matrix metalloproteinases, vascular remodeling, and vascular disease. Adv. Pharmacol..

[16] Zhang B., Henney A., Eriksson P., Hamsten A., Watkins H., Ye S. (1999). Genetic variation at the matrix metalloproteinase-9 locus on chromosome 20q12.2-13.1. Hum. Genet..

[17] Blankenberg S., Rupprecht H.J., Poirier O., Bickel C., Smieja M., Hafner G., Meyer J., Cambien F., Tiret L. (2003). Plasma concentrations and genetic variation of matrix metalloproteinase 9 and prognosis of patients with cardiovascular disease. Circulation.

[18] Opstad T.B., Pettersen A.-A.R., Weiss T.W., Åkra S., Øvstebø R., Arnesen H., Seljeflot I. (2012). Genetic variation, gene-expression and circulating levels of matrix metalloproteinase-9 in patients with stable coronary artery disease. Clin. Chim. Acta.

[19] Hassanzadeh-Makoui R., Razi B., Aslani S., Imani D., Tabaee S.S. (2020). The association between Matrix Metallo-proteinases-9 (MMP-9) gene family polymorphisms and risk of Coronary Artery Disease (CAD): A systematic review and meta-analysis. BMC Cardiovasc. Disord..

[20] Koh Y.S., Chang K., Kim P.J., Seung K.B., Baek S.H., Shin W.S., Lim S.H., Kim J.H., Choi K.B. (2008). A close relationship between functional polymorphism in the promoter region of matrix metalloproteinase-9 and acute myocardial infarction. Int. J. Cardiol..

[21] Rodríguez-Pérez J., Vargas-Alarcón G., Posadas-Sánchez R., Zagal-Jiménez T., Ortíz-Alarcón R., Valente-Acosta B., Tovilla-Zárate C., Nostroza-Hernández C., Pérez-Méndez O., Pérez-Hernández N. (2016). rs3918242 MMP9 gene polymorphism is associated with myocardial infarction in Mexican patients. Genet. Mol. Res..

[22] Gajewska B., Śliwińska-Mossoń M. (2022). Association of MMP-2 and MMP-9 polymorphisms with diabetes and pathogenesis of diabetic complications. Int. J. Mol. Sci..

[23] Buraczynska M., Zakrocka I. (2021). Arginase gene polymorphism increases risk of diabetic retinopathy in type 2 diabetes mellitus patients. J. Clin. Med..

[24] American Diabetes Association (2018). Chapter 2. Classification and diagnosis of diabetes: Standards of medical care in diabetes—2018. Diabetes Care.

[25] Nie S.W., Wang X.F., Tang Z.C. (2014). Correlations between MMP-2 / MMP-9 promoter polymorphisms and ischemic stroke. Int. J. Clin. Exp. Med..

[26] Gorący I., Grudniewicz S., Safranow K., Ciechanowicz A., Jakubiszyn P., Gorący A., Brykczyński M. (2020). Genetic polymorphisms of *MMP1*, *MMP9*, *COL1A1*, and *COL1A2* in polish patients with thoracic aortopathy. Dis. Markers.

[27] Rawshani A., Rawshani A., Rawshani A., Franzén S., Sattar N., Eliasson B., Svensson A.-M., Zethelius B., Miftaraj M., McGuire D.K. (2018). Risk factors, mortality, and cardiovascular outcomes in patients with type 2 diabetes. N. Engl. J. Med..

[28] Singh K., Agrawal N.K., Gupta S.K., Singh K. (2013). A functional single nucleotide polymorphism -1562 C>T in the matrix metalloproteinase-9 promoter is associated with type 2 diabetes and diabetic foot ulcers. Int. J. Low. Extrem. Wounds.

[29] Zhang Z., Wu X., Cai T., Gao W., Zhou X., Zhao J., Yao J., Shang H., Dong J., Liao L. (2015). Matrix metalloproteinase 9 gene promoter (rs3918242) mutation reduces risk of diabetic microvascular complications. Int. J. Environ. Res. Public Health.

[30] Buraczynska K., Kurzepa J., Ksiazek A., Buraczynska M., Rejdak K. (2015). Matrix metalloproteinase-9 (MMP-9) gene poymorphism in stroke patients. Neuromol. Med..

[31] Goracy J., Goracy I., Brykczyński M., Peregud-Pogorzelska M., Naruszewicz M., Ciechanowicz A. (2003). [The C(-1562)T polymorphism in the promoter of the matrix metalloproteinase-9 (MMP-9) gene and coronary atherosclerosis]. Pol. Arch. Med. Wewn..

[32] Watson C., Spiers J.P., Waterstone M., Russell-Hallinan A., Gallagher J., McDonald K., Ryan C., Gilmer J., Ledwidge M. (2021). Investigation of association of genetic variant rs3918242 of matrix metalloproteinase-9 with hypertension, myocardial infarction and progression of ventricular dysfunction in Irish Caucasian patients with diabetes: A report from the STOP-HF follow-up programme. BMC Cardiovasc. Disord..

[33] Opstad T.B., Arnesen H., Pettersen A., Seljeflot I. (2014). The MMP-9 -1562 C/T Polymorphism in the presence of metabolic syndrome increases the risk of clinical events in patients with coronary artery disease. PLoS ONE.

[34] Boshev M., Stankovic S., Panov S., Josifovska S., Georgiev A., Poposka L., Pejkov H. (2023). Association of the polymorphism rs3918242 of the matrix metalloproteinase-9 gene with coronary artery disease in a younger population. Contrib. Sec. Med. Sci..

[35] Qin L., Qin G., Shi X., Wang A., Zuo H. (2016). Association between matrix metalloproteinase-9 rs391824 polymorphism and development of coronary artery disease in a Chinese population. Genet. Mol. Res..

[36] Kaplan R.C., Smith N.L., Zucker S., Heckbert S.R., Rice K., Psaty B.M. (2008). Matrix metalloproteinase-3 (MMP-3) and MMP-9 genes and risk of myocardial infarction, ischemic stroke, and hemorrhagic stroke. Atherosclerosis.

[37] Mahmoodi K., Kamali K., Karami E., Soltanpour M.S. (2017). Plasma concentration, genetic variation, and expression levels of matrix metalloproteinase 9 in Iranian patients with coronary artery disease. J. Res. Med. Sci..

[38] Basia D., Gupta M.D., Kunal S., Muheeb G., Girish M., Bansal A., Batra V., Yusuf J., Mukhopadhyay S., Tyagi S. (2022). Matrix metalloproteinases and their gene polymorphism in young ST-segment elevation myocardial infarction. Indian Heart J..

[39] Morgan A.R., Zhang B., Tapper W., Collins A., Ye S. (2003). Haplotype analysis of MMP-9 gene in relation to coronary artery disese. J. Mol. Med..

[40] Põllänen P.J., Karhunen P.J., Mikkelsson I., Laippala P., Perola M., Pentilla A., Mattila K.M., Koivula T., Lehtimäki T. (2001). Coronary artery complicated lesion area is related to functional polymorphism of matrix metalloproteinase-9 gene: An autopsy study. Arterioscler. Thromb. Vasc. Biol..

[41] Paulus W.J., Tschõppe C. (2013). A novel paradigm for heart failure with preserved ejection fraction: Comorbidities drive myocardial dysfunction and remodeling through coronary microvascular endothelial inflammation. J. Am. Coll. Cardiol..

